# Functional Imaging to Guide Network-Based TMS Treatments: Toward a Tailored Medicine Approach in Alzheimer’s Disease

**DOI:** 10.3389/fnins.2021.687493

**Published:** 2021-07-05

**Authors:** Chiara Bagattini, Debora Brignani, Sonia Bonnì, Giulia Quattrini, Roberto Gasparotti, Michela Pievani

**Affiliations:** ^1^Neurophysiology Lab, IRCCS Istituto Centro San Giovanni di Dio Fatebenefratelli, Brescia, Italy; ^2^Non Invasive Brain Stimulation Unit/Department of Behavioral and Clinical Neurology, Santa Lucia Foundation IRCCS, Rome, Italy; ^3^Laboratory Alzheimer’s Neuroimaging and Epidemiology, IRCCS Istituto Centro San Giovanni di Dio Fatebenefratelli, Brescia, Italy; ^4^Department of Molecular and Translational Medicine, University of Brescia, Brescia, Italy; ^5^Neuroradiology Unit, ASST Spedali Civili Hospital, University of Brescia, Brescia, Italy

**Keywords:** Alzheimer’s disease, functional brain networks, resting-state fMRI, connectivity, tailored treatment, repetitive transcranial magnetic stimulation

## Abstract

A growing number of studies is using fMRI-based connectivity to guide transcranial magnetic stimulation (TMS) target identification in both normal and clinical populations. TMS has gained increasing attention as a potential therapeutic strategy also in Alzheimer’s disease (AD), but an endorsed target localization strategy in this population is still lacking. In this proof of concept study, we prove the feasibility of a tailored TMS targeting approach for AD, which stems from a network-based perspective. Based on functional imaging, the procedure allows to extract individual optimal targets meanwhile accounting for functional variability. Single-subject resting-state fMRI was used to extract individual target coordinates of two networks primarily affected in AD, the default mode and the fronto-parietal network. The localization of these targets was compared to that of traditional group-level approaches and tested against varying degrees of TMS focality. The distance between individual fMRI-derived coordinates and traditionally defined targets was significant for a supposed TMS focality of 12 mm and in some cases up to 20 mm. Comparison with anatomical labels confirmed a lack of 1:1 correspondence between anatomical and functional targets. The proposed network-based fMRI-guided TMS approach, while accounting for inter-individual functional variability, allows to target core AD networks, and might thus represent a step toward tailored TMS interventions for AD.

## Introduction

Through the repeated delivery of short-lived magnetic fields over the scalp, repetitive transcranial magnetic stimulation (rTMS) is able to induce long-lasting changes of cortical excitability, which resemble long-term potentiation or long-term depression-like mechanisms, depending on the stimulation parameters (Wassermann et al., 2008). Robust evidence proves that TMS acts beyond the site of stimulation, affecting the connectivity of the stimulated networks (Ruff et al., 2009; Siebner et al., 2009; Fox et al., 2012b), thus rising considerable interest for its therapeutic application across a range of diseases with distributed network pathology (Fox et al., 2012a; Lefaucheur et al., 2014).

A growing number of studies have focused on brain connectivity as a promising approach to guide TMS treatment. Functional magnetic resonance imaging (fMRI)-based network connectivity has been already successfully used for the identification of TMS target in healthy young (Santarnecchi et al., 2018; Momi et al., 2020; Ozdemir et al., 2020) and elderly participants (Wang et al., 2014; Nilakantan et al., 2019), as well as in psychiatric patients (Hoffman et al., 2007; Fox et al., 2012a), but not in neurodegenerative disorders such as Alzheimer’s Disease (AD).

While rTMS has gained increasing attention as a potential treatment for AD (Weiler et al., 2020), evidence regarding its clinical efficacy is feeble and key issues remain before its clinical application (Lefaucheur et al., 2020). The majority of previous rTMS studies individuated the target areas through coarse procedures, such as rule of thumb, EEG electrode system, group-averaged coordinates or anatomical landmarks (please refer to Table 1 for an overview of methods adopted in previous studies).

**TABLE 1:** Summary of the target areas and localization methods adopted in previous rTMS interventions in AD patients.

Study	Target area(s)	Localization method
***5-cm rule***		
Ahmed et al., 2012	Left and right DLPFC	5 cm rostral to optimal site for motor threshold production in the first dorsal interosseous
Haffen et al., 2012	Left DLPFC	5 cm anterior and parasagittal from the hand area
Drumond Marra et al., 2015	Left DLPFC	5 cm in a parasagittal plane parallel to the point to maximum stimulation of the short abductor of the thumb
***Electrode position(s) according to the International 10–20 EEG System***		
Zhao et al., 2017	Left and right parietal and posterior-temporal areas	P3, P4, T5, T6
Alcalá-Lozano et al., 2018	Broca, Wernicke, right and left DLPFC, right and left pSAC	Left DLPFC: electrode not defined; other regions: localization method not defined
Turriziani et al., 2019	Right DLPFC	F4
Bagattini et al., 2020	Left DLPFC	F3
***Group-average coordinates (mean Tailarach coordinates)***		
Cotelli et al., 2010	Left DLPFC (BA 8/9)	*x* = −35, *y* = 24, *z* = 48
Cotelli et al., 2012	Left IPL	*x* = −44, *y* = −51, *z* = 43
***Individual anatomical landmarks***		
Bentwich et al., 2011	Broca, Wernicke, right and left DLPFC, right and left pSAC	Identified by neuroradiologist on individual MRI scans
Rabey et al., 2013	Broca, Wernicke, right and left DLPFC, right and left pSAC	Identified by neuroradiologist on individual MRI scans
Rabey and Dobronevsky, 2016	Broca, Wernicke, right and left DLPFC, right and left pSAC	Not better defined
Lee et al., 2016	Broca, Wernicke, right and left DLPFC, right and left pSAC	Identified by neuroradiologist on individual MRI scans
Nguyen et al., 2018	Broca, Wernicke, right and left prefrontal cortex, right and left parietal cortex	Identified by the Neuronix neuronavigation system based on the individual MRI.
Koch et al., 2018	Precuneus	Individual T1-weighted MRI volumes were used as anatomical reference
Sabbagh et al., 2020	Broca, Wernicke, right and left DLPFC, right and left parietal cortex	Brain regions were marked in individual MRI scan by projecting the relevant brain region onto the scalp

These approaches, however, do not account for the functional organization of the brain and the synaptic dysfunction affecting specific networks in AD. In particular, AD is associated with the disruption of several large-scale networks, of which two play a central role in cognition, the Default Mode Network (DMN) and the Fronto-Parietal Network (FPN) (Agosta et al., 2012; Pievani et al., 2014). The DMN is medially anchored to the posterior cingulate cortex/precuneus and ventromedial prefrontal cortex, and to the bilateral parietal (inferior parietal lobule – IPL, which include the angular and inferior parietal gyri), temporal (lateral temporal cortex and hippocampi), and frontal cortex (dorsolateral prefrontal cortex – DLPFC, roughly corresponding to the superior frontal gyrus). The FPN includes the bilateral DLPFC (middle frontal gyrus) and parietal (superior parietal gyrus) cortex. Due to their crucial role in modulating cognition in AD, targeting these functional networks might represent a valid option for rTMS treatments in this population. The clinical promise of stimulating AD-core networks such as DMN is demonstrated by a recent study showing an improvement in memory by targeting the precuneus (Koch et al., 2018). Moreover, although some of the previous rTMS studies might have stimulated regions belonging to these networks (i.e., DLPFC node of the FPN, IPL node of the DMN; Lefaucheur et al., 2020), this remains speculative lacking a direct assessment with neuroimaging.

Given the potential value of tailored network-based rTMS intervention for neurocognitive and psychiatric diseases, here we demonstrate the feasibility of a TMS approach that uses resting-state fMRI to identify and target functionally, patho-physiologically and clinically relevant AD networks at the individual level. This strategy is compared to traditional approaches for target localization.

## Materials and Equipment

Magnetic resonance imaging (MRI) scans were acquired on a 3T Siemens Skyra scanner equipped with a 64-channels head-neck coil at the Neuroradiology Unit, Spedali Civili Hospital (Brescia, Italy). Multiband accelerated resting-state fMRI (rs-fMRI) (TR = 1000 ms, TE = 27 ms, flip angle = 60°, voxel size = 2.1 mm isotropic, 70 slices, 600 volumes) and 3D T1-weighted (TR = 2300 ms, TE = 2 ms, flip angle = 9°, voxel size = 1 mm isotropic, 176 slices) scans were collected.

## Methods

**FIGURE 1:**
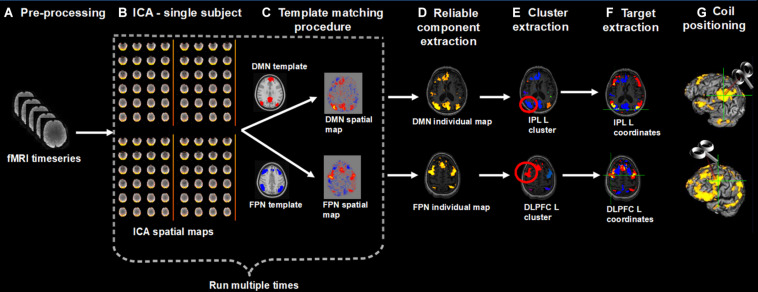
Overview of the procedure for the identification and selection of individual DMN and FPN targets based on rs-fMRI; **(A)** Collected rs-fMRI data were pre-processed removing the first time-points, correcting motion, and susceptibility-induced distortions; **(B)** DMN and FPN were extracted from individual rs-fMRI scans using independent component analysis (ICA); **(C)** Networks of interest (in MNI space) were identified using a template matching procedure; **(B,C)** were repeated multiple times; **(D)** The most reliable components were identified and back-transformed to subjects’ native T1 space; **(E)** Each network was decomposed into clusters and the largest cluster in the left IPL and left DLPFC was identified, for the DMN and FPN, respectively; **(F)** The peaks (local maxima) within these clusters were extracted and the final individual TMS targets were selected according to the following criteria: (i) location specific to the network of interest, i.e., coordinates falling within the spatial maps of both DMN and FPN (yellow areas) were excluded (blue = DMN, red = FPN); (ii) being on a cortical gyrus and not on a sulcus (i.e., overlap with GM); (iii) representing the shortest perpendicular path between scalp and cortex; **(G)** TMS coil was positioned through a neuronavigation system to target the selected DMN and FPN coordinates.

We developed a pipeline to extract individual targets from rs-fMRI data for the stimulation of the DMN and FPN. We choose as targets the IPL node of the DMN and the DLPFC node of the FPN, since these targets are similar to those traditionally stimulated by previous rTMS studies in AD. The procedure, however, can be applied to other DMN and FPN regions as well (e.g., the lateral temporal node of the DMN, the DLPFC node of the DMN, the superior parietal gyrus of the FPN). Medial nodes such as the posterior cingulate cortex or the medial prefrontal cortex were not considered since these regions are difficult to reach with traditional coils. Moreover, we focused on the left hemisphere since a recent meta-analysis revealed that the effects of rTMS at the DLPFC are lateralized: high-frequency rTMS (i.e., the most adopted rTMS protocol) over the left hemisphere significantly improved memory functions (Chou et al., 2019). However, the procedure can be applied to extract contralateral targets as well. First, rs-fMRI data are pre-processed according to standard steps (removal of the first volumes for signal equilibrium, motion correction, susceptibility-induced distortions correction). Then, independent component analysis (ICA) is used to decompose the fMRI data into different spatial and temporal components. The spatial maps are transformed to standard MNI space to identify the networks of interest (in our case, the DMN and FPN) according to a template matching procedure. Alternatively, the components can be identified based on visual inspection. The ICA step is repeated multiple times to check for the reliability of the components and the most reliable components are selected. The resulting spatial maps are expressed as t-statistics or z-statistics, higher values indicating a higher degree of activation within the component or correlation with the time series. The spatial maps are decomposed into clusters; the largest clusters in the left IPL and left DLPFC areas are identified based on visual inspection; the cluster peaks (e.g., local maxima) are extracted as potential targets. The final individual TMS targets are selected according to the following criteria: (i) location specific to the network of interest, i.e., coordinates falling within the spatial maps of both DMN and FPN are excluded; (ii) being on a cortical gyrus and not on a sulcus (i.e., overlap with GM); (iii) representing the shortest perpendicular path between scalp and cortex. Finally, to stimulate the selected DMN and FPN coordinates, the TMS coil is positioned through a neuronavigation system. The entire procedure is summarized in Figure 1.

We tested this procedure in a sample of mild AD patients [*n* = 13; age: 73.54 years (min 56 – max 85); seven females; MMSE: 21.23 (min 17 – max 25)] (McKhann et al., 2011) recruited between June 2019 and April 2021 at the IRCCS Fatebenefratelli (Brescia, Italy) and at the IRCCS Santa Lucia (Rome, Italy) in the context of an ongoing randomized controlled clinical trial (GR-2016-02364718; NCT04263194). The study was approved by the local ethics committee and participants signed a written informed consent.

rs-fMRI data pre-processing was carried out using the FMRIB’s Software Library (FSL; Smith et al., 2004)^1^. After removal of the first ten time-points, motion correction was carried out with FLIRT (part of FSL) and correction of susceptibility-induced distortions with TOPUP (part of FSL) (Andersson et al., 2003). ICA was applied with Melodic (Beckmann and Smith, 2004)^2^. Melodic processing included high-pass temporal filtering (0.01 Hz), smoothing with a 4 mm FWHM filter, affine transformation of EPI images to native T1 images and non-linear warping of T1 images to standard MNI space. The number of components was automatically estimated by Melodic. The template matching procedure was applied using previously published templates (Shirer et al., 2012). For reliability assessment, Melodic was run 10 times and the spatial maps most frequently classified as “DMN” or “FPN” were retained. The selected DMN and FPN spatial maps were then back-transformed to subjects’ native T1 space using Melodic transformations. FSL’s *cluster* routine was used to decompose each network into clusters and to derive the peak (local maxima) within each cluster (left IPL and left DLPFC). The local maxima were overlaid onto the native T1 scan and the final targets were selected according to the above described criteria.

To check for the anatomical-functional correspondence of each target, the anatomical atlas label (AAL; Tzourio-Mazoyer et al., 2002) was used to label individual coordinates with the corresponding anatomical region.

The distance between individual rs-fMRI derived and traditional anatomical coordinates was computed as follows. Individual coordinates in native space were transformed to MNI space using the affine and non-linear warping estimated by Melodic. The Euclidean distance was used to compute the distance from group-level left IPL and DLPFC coordinates reported in previous TMS studies (Herwig et al., 2003; Cotelli et al., 2010, 2012; Fox et al., 2013). Coordinates in Talairach space were transformed to MNI space using a non-linear transformation (Lacadie et al., 2008). For studies using the Brett or Lancaster transformation to derive Talairach coordinates, we used the inverse Brett/Lancaster transformation to obtain the original MNI coordinates. One-sample Wilcoxon test was used to assess whether the distance between individual and traditional coordinates exceeded two threshold’s levels, assuming a spatial extent of rTMS-induced activation of 12 mm (conservative threshold; Fox et al., 2013) and 20 mm (lenient threshold).

Finally, we compared the precision of our approach with traditional approaches testing (i) the sensitivity of group-level IPL and DLPFC coordinates to DMN and FPN spatial maps, respectively (i.e., how frequently group-level coordinates fell into the expected network), and (ii) the selectivity of this relationship (i.e., how frequently a coordinate falling into one network also fell into the other). Group-level coordinates were overlaid onto the individual spatial maps of the DMN and FPN before computing the above frequencies.

### Generalization to Healthy Elderly Population

In order to provide evidence on the generalization of the proposed individual network-based targeting approach to other populations, the same procedure applied in mild AD patients was tested in a sample of healthy elderly controls [*n* = 8; age: 66.38 years (min 60 – max 75); three females; MMSE: 29.75 (min 28 – max 30)] recruited at the IRCCS Fatebenefratelli (Brescia, Italy) between February 2021 and April 2021.

### Validation With Seed-Connectivity Analysis

In order to assess the validity of the individual rs-fMRI coordinates obtained with our approach, seed connectivity analysis was computed. First, individual coordinates were used as seed for a whole-brain connectivity analysis. Thereafter, group-level coordinates were used as seeds. We then tested whether the seed-connectivity derived maps best matched to the individual DMN or FPN spatial map derived from ICA. For the seed-based correlation analysis, we created spherical ROIs (6 mm radius) centered on the target coordinates (in native T1 space for the TMS targets and in MNI space for the group-level coordinates) and transformed them to native EPI space. We then computed the correlation coefficients between the time-series within each seed and the time-series of all the other brain voxels. Finally, a template-matching procedure was used to test whether each seed-connectivity map best matched to the individual DMN or FPN spatial map ICA-derived.

## Results

**FIGURE 2:**
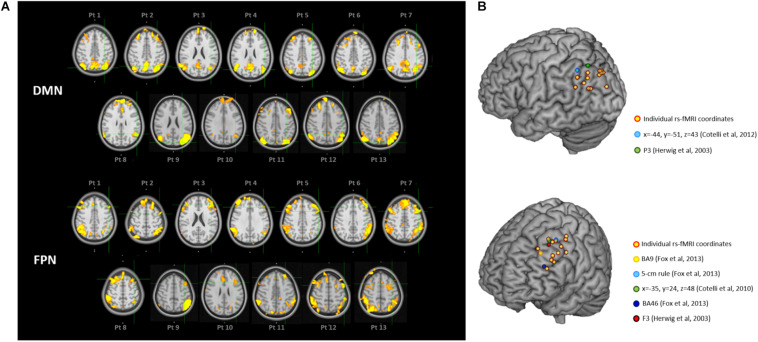
**(A)** Location of the individual targets (overlaid onto the standard MNI template) for default mode network (DMN) stimulation **(top panel)** and frontoparietal network (FPN) stimulation **(bottom panel)** in thirteen AD patients. Images are shown in radiological convention (left denotes right). The individual targets (green cross) were extracted from each subject’s 3T rs-fMRI data using ICA. The DMN targets correspond to the left IPL cluster, the FPN targets to the left DLPFC cluster. The individual DMN and FPN maps are shown in orange-yellow. The targets were defined in subjects’ native T1 space and back-transformed to the standard MNI space for computation and visualization purposes; **(B)** 3D render showing the individual targets (red-yellow) overlaid onto the standard MNI template. For the DMN, green target corresponds to P3 (Herwig et al., 2003), and light-blue to IPL (Cotelli et al., 2012). For the FPN, yellow target corresponds to DLPFC BA9 (Fox et al., 2013), light-blue to DLPFC-5 cm rule (Fox et al., 2013), blue to DLPFC BA46 (Fox et al., 2013), red to F3 (Herwig et al., 2003), green to DLPFC BA8/9 (Cotelli et al., 2010). DMN, default mode network; FPN, fronto-parietal network; BA8/9, Broadmann areas 8 and 9; BA9, Broadmann area 9; BA46, Broadmann area 46; IPL, inferior parietal lobule; DLPFC, dorsolateral prefrontal cortex.

Individual targets are shown relative to their network in Figure 2A, and their position is depicted in Figure 2B compared to the group-level coordinates (all coordinates are reported in MNI space). The median distance between individual IPL coordinates was 20.39 mm (interquartile range: 14.70–26.31 mm) and between individual DLPFC coordinates was 21.68 mm (interquartile range: 17.20–27.28 mm). When using the anatomical atlas label (AAL; Tzourio-Mazoyer et al., 2002) to localize our IPL coordinates, 5 out of 13 cases corresponded to or were close to the angular gyrus (AG), five to the middle occipital gyrus (MOG), two to the inferior parietal gyrus (IPL), and one was borderline between the latter two regions (Table 2). The median distance between individual fMRI-derived and group-level IPL coordinates was >20 mm for both the studies considered (Herwig et al., 2003; Cotelli et al., 2012). However, the distance between individual fMRI-derived and P3 coordinates (Herwig et al., 2003) significantly exceeded rTMS focality when considering the 12 mm threshold (*p* = 0.0002), but not the 20 mm threshold (*p* = 0.342; Table 2). When compared with the IPL coordinates used in Cotelli et al. (2012), the distance significantly exceeded both rTMS focalities (all *p*’s < 0.05; Table 2).

**TABLE 2:** Individual coordinates (reported in standard MNI space) of the two targets (the left IPL node of the DMN and the left DLPFC node of the FPN) obtained with the individual rs-fMRI guided approach in the sample of AD patients.

	DMN – left IPL				FPN – left DLPFC						
	Individual rs-fMRI coordinates	AAL	Distance (mm) from P3	Distance (mm) from IPL	Individual rs-fMRI coordinates	AAL	Distance (mm) from BA9	Distance (mm) from BA46	Distance (mm) from 5 cm rule	Distance (mm) from F3	Distance (mm) from BA8/9
Pt 1	−34 −80 44	IPL	16.16	27.93	−52 24 40	MFG	21.26	24.04	18.60	16.16	20.10
Pt 2	−38 −66 42	AG	9.00	14.00	−48 4 56	PCG	39.45	48.44	13.64	24.60	22.00
Pt 3	−44 −68 24	MOG	27.73	26.08	−46 38 22	MFG	20.59	4.69	38.13	30.15	35.44
Pt 4	−52 −72 26	AG	29.27	28.07	−48 32 32	MFG	15.62	12.08	27.17	20.12	25.38
Pt 5	−36 −82 42	MOG	18.47	29.39	−58 16 40	MFG/PCG	31.11	32.34	20.83	23.35	25.77
Pt 6	−34 −82 44	IPL/MOG	17.92	29.80	−50 32 38	MFG	15.36	16.91	23.79	17.12	22.18
Pt 7	−40 −66 36	AG	15.13	16.12	−50 16 46	MFG	26.76	33.20	10.86	15.13	16.37
Pt 8	−56 −54 28	AG	31.58	21.63	−40 28 56	MFG	19.29	33.85	13.64	8.77	8.25
Pt 9	−32 −88 24	MOG	35.34	42.24	−38 18 50	MFG	22.45	4.69	4.69	7.28	4.90
Pt 10	−42 −62 30	AG	21.75	18.00	−42 20 26	IFG	23.58	26.50	26.50	22,91	26.76
Pt 11	−38 −80 40	MOG	17.80	27.35	−30 36 44	MFG	7.48	25.02	25.02	14.18	17.20
Pt 12	−54 −56 40	IPL	21.84	11.83	−52 10 54	MFG/PCG	35.16	12.25	12.25	21.38	20.10
Pt 13	−34 −84 42	MOG	20.52	31.87	−38 12 50	MFG	27.93	4.69	4.69	13.15	10.39
Median (IQR)	**20.39 (14.70– 26.31)**		**20.52 (17.80–27.73)**	**27.35 (18.00–29.39)**	**21.68 (17.20–27.28)**		**22.45 (19.29–27.93)**	**32.34 (21.12–34.50)**	**18.60 (12.25–25.02)**	**17.12 (14.18–22.91)**	**20.10 (16.37–25.38)**
*p (12 mm threshold)*	<0.0001*		0.0002*	0.0002*	<0.0001*		0.0006*	0.0006*	0.018*	0.005*	0.005*
*p (20 mm threshold)*	0.353		0.342	0.029*	0.018*		0.095	0.024*	0.758	0.863	0.472

The corresponding anatomical region is provided based on the Anatomical atlas label (AAL), and the average distance between individual coordinates and group-level coordinates is provided. Results of one-sample Wilcoxon tests (*p*-values) assessing the null hypothesis that the distance between individual and group-level coordinates is below 12 mm and 20 mm are reported (*significant difference). DMN, default mode network; FPN, fronto-parietal network; BA8/9, Broadmann areas 8 and 9; BA9, Broadmann area 9; BA46, Broadmann area 46; IPL, inferior parietal lobule; DLPFC, dorsolateral prefrontal cortex; AG, angular gyrus; IFG, inferior frontal gyrus; MFG, middle frontal gyrus; MOG, middle occipital gyrus; PCG, precentral gyrus; IQR, interquartile range. Bold numbers depict median and IQR scores.

The sensitivity of group-level IPL coordinates to individual DMN spatial maps was 46% in the best case (Herwig et al., 2003) while selectivity was generally low (>67% of the coordinates falling into the DMN also fell within the FPN) (Table 3).

**TABLE 3:** Correspondence between group-level IPL and DLPFC coordinates and individual DMN and FPN maps.

Group-level coordinates	Sensitivity	Selectivity
**DMN**		
P3 (Herwig et al., 2003)	46%	33%
IPL (Cotelli et al., 2012)	31%	0%
**FPN**		
F3 (Herwig et al., 2003)	62%	50%
BA8/9 (Cotelli et al., 2010)	46%	33%
5-cm rule (Fox et al., 2013)	54%	71%
BA9 (Fox et al., 2013)	23%	33%
BA46 (Fox et al., 2013)	15%	50%

*As a reference, individual fMRI-derived coordinates have a sensitivity and selectivity of 100%. DMN, default mode network; FPN, fronto-parietal network; BA8/9, Broadmann areas 8 and 9; BA9, Broadmann area 9; BA46, Broadmann area 46; IPL, inferior parietal lobule; DLPFC, dorsolateral prefrontal cortex.*

Dorsolateral prefrontal cortex coordinates were localized in the middle frontal gyrus (MFG) in 9 out of 13 cases (69.3% of cases), in the precentral gyrus (PCG) in one case, borderline between the two in two cases, and in the inferior frontal gyrus (IFG) in one case (Table 2). The distance between individual fMRI-derived and group-level DLPFC coordinates (Herwig et al., 2003; Cotelli et al., 2010; Fox et al., 2013) was significant for all group-level coordinates at the 12 mm threshold (all *p*’s < 0.05), but not at the 20 mm threshold (all *p*’s > 0.10), except for DLPFC BA46 (Fox et al., 2013) which significantly exceeded the threshold (*p* = 0.024; Table 2). Group-level DLPFC coordinates most sensitive to FPN spatial maps were F3 coordinates (62% of coordinates falling into the FPN), followed by 5 cm-rule (54%), and DLPFC BA8/9 (46%) coordinates. The selectivity of these coordinates, however, was relatively good only for the 5 cm rule (71% of the coordinates being specific for the FPN), and low for the remaining group level coordinates (50% for F3 and BA46 and 67% for BA8/9 and BA9 of cases also falling into the DMN) (Table 3).

### Generalization to Healthy Elderly Population

**FIGURE 3:**
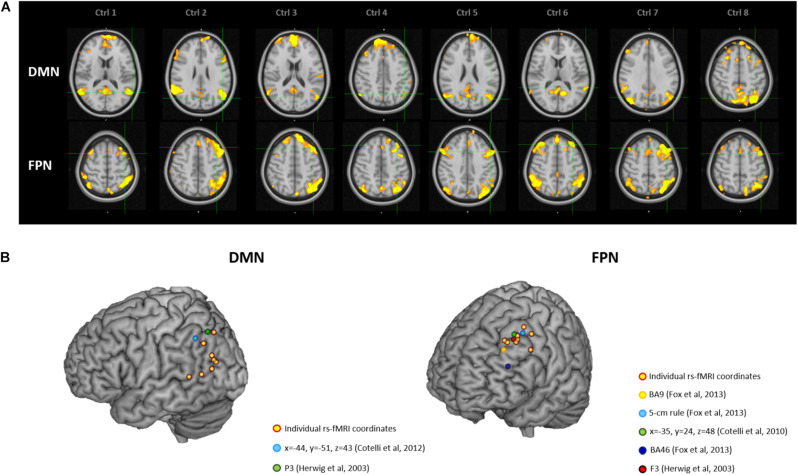
**(A)** Location of the individual targets (overlaid onto the standard MNI template) for default mode network (DMN) stimulation **(top panel)** and frontoparietal network (FPN) stimulation **(bottom panel)** in eight healthy elderly controls. Images are shown in radiological convention (left denotes right). **(B)** 3D render showing the individual targets (red-yellow) overlaid onto the standard MNI template. For the DMN, green target corresponds to P3 (Herwig et al., 2003), and light-blue to IPL (Cotelli et al., 2012). For the FPN, yellow target corresponds to DLPFC BA9 (Fox et al., 2013), light-blue to DLPFC-5 cm rule (Fox et al., 2013), blue to DLPFC BA46 (Fox et al., 2013), red to F3 (Herwig et al., 2003), green to DLPFC BA8/9 (Cotelli et al., 2010). DMN, default mode network; FPN, fronto-parietal network; BA8/9, Broadmann areas 8 and 9; BA9, Broadmann area 9; BA46, Broadmann area 46; IPL, inferior parietal lobule; DLPFC, dorsolateral prefrontal cortex.

Figure 3 depicts individual IPL and DLPFC targets relative to their network (Figure 3A), with their position compared to the coordinates reported in the literature (Figure 3B), in the sample of healthy elderly controls. Individual rs-fMRI coordinates and their comparison with group-level coordinates are shown in Table 4. The median distance between individual IPL coordinates was 21.35 mm (interquartile range: 13.24–30.30 mm) and between individual DLPFC coordinates was 15.75 mm (interquartile range: 10.84–21.32 mm). When using the anatomical atlas label (AAL; Tzourio-Mazoyer et al., 2002) to localize our IPL coordinates, four out of eight cases corresponded to the AG, two to the MTG, one to the IPL, and one was borderline between the MTG and the MOG (Table 4). The median distance between individual fMRI-derived and group-level IPL coordinates was >28 mm for both the studies considered (Herwig et al., 2003; Cotelli et al., 2012). Consistently with the results in AD patients, these distances significantly exceeded rTMS focality when considering both the conservative (*p* = 0.012 and *p* = 0.008, respectively) and the lenient threshold (*p* = 0.054 and *p* = 0.027, respectively; Table 5).

**TABLE 4:** Individual coordinates (reported in standard MNI space) of the two targets (the left IPL node of the DMN and the left DLPFC node of the FPN) obtained with the individual rs-fMRI guided approach in the sample of healthy elderly controls.

	DMN – left IPL				FPN – left DLPFC						
	Individual rs-fMRI coordinates	AAL	Distance (mm) from P3	Distance (mm) from IPL	Individual rs-fMRI coordinates	AAL	Distance (mm) from BA9	Distance (mm) from BA46	Distance (mm) from 5 cm rule	Distance (mm) from F3	Distance (mm) from BA8/9
Ctrl 1	−60 −60 14	MTG	43.46	36.28	−40 12 58	MFG	31.87	44.11	6.78	16.52	12.33
Ctrl 2	−50 −72 26	AG	28.37	27.57	−42 26 46	MFG	14.70	25.88	12.57	4.58	9.38
Ctrl 3	−54 −72 26	AG	36.89	33.76	−42 24 48	MFG	17.20	28.67	9.90	4.12	7.48
Ctrl 4	−48 −62 42	AG	14.04	9.80	−36 32 46	MFG	8.49	24.21	18.71	7.55	11.66
Ctrl 5	−50 −74 24	MTG/MOG	30.61	30.33	−52 16 38	MFG	27.28	29.29	17.83	19.42	22.09
Ctrl 6	−62 −48 12	MTG	49.20	38.94	−34 34 48	MFG	9.17	26.04	20.64	9.85	12.81
Ctrl 7	−56 −70 32	AG	26.48	24.41	−42 24 52	MFG	19.39	31.97	9.06	5.74	6.32
Ctrl 8	−36 −72 50	IPL	6.40	20.10	−48 10 52	PCG	32.74	41.30	8.60	18.47	16.97
Median (IQR)	**21.35 (13.24– 30.30)**		**29.49 (20.26–40.18)**	**28.95 (22.26–35.02)**	**15.75 (10.84–21.32)**		**18.30 (11.94–29.58)**	**28.98 (25.96–36.64)**	**11.23 (8.83–18.27)**	**8.70 (5.16– 17.50)**	**12.00 (8.43–14.89)**
*p (12 mm threshold)*	<0.0001*		0.012*	0.008*	0.0098*		0.039*	0.004*	0.320	0.680	0.527
*p (20 mm threshold)*	0.172		0.054#	0.027*	0.997		0.473	0.004*	0.996	1.000	0.996

The corresponding anatomical region is provided based on the Anatomical atlas label (AAL) and the average distance between individual coordinates and group-level coordinates is provided. Results of one-sample Wilcoxon tests (*p*-values) assessing the null hypothesis that the distance between individual and group-level coordinates is below 12 mm and 20 mm are reported (*significant difference; #trend toward significance). DMN, default mode network; FPN, fronto-parietal network; BA8/9, Broadmann areas 8 and 9; BA9, Broadmann area 9; BA46, Broadmann area 46; IPL, inferior parietal lobule; DLPFC, dorsolateral prefrontal cortex; AG, angular gyrus; IFG, inferior frontal gyrus; MFG, middle frontal gyrus; MTG, middle temporal gyrus; MOG, middle occipital gyrus; PCG, precentral gyrus; IQR, interquartile range. Bold numbers depict median and IQR scores.

**TABLE 5:** Correspondence between the individual ICA maps of the DMN and FPN and the seed connectivity maps obtained using (A) the individual TMS targets or (B) the group-level coordinates as seeds.

DMN – left IPL			FPN – left DLPFC		
	Seed	Match with individual DMN ICA		Seed	Match with individual FPN ICA
A	Individual rs-fMRI coordinates	13/13 (100%)	A	Individual rs-fMRI coordinates	11/13 (85%)
B	IPL P3 (Herwig et al., 2003)	8/13 (62%)	B	5-cm rule (Fox et al., 2013)	7/13 (54%)
B	IPL (Cotelli et al., 2012)	4/13 (31%)	B	BA9 (Fox et al., 2013)	9/13 (69%)
			B	BA46 (Fox et al., 2013)	5/13 (38%)
			B	F3 (Herwig et al., 2003)	5/13 (38%)
			B	BA8/9 (Cotelli et al., 2010)	5/13 (38%)

*Correspondence was assessed with a template matching procedure. DMN, default mode network; FPN, fronto-parietal network; BA8/9, Broadmann areas 8 and 9; BA9, Broadmann area 9; BA46, Broadmann area 46; IPL, inferior parietal lobule; DLPFC, dorsolateral prefrontal cortex.*

Dorsolateral prefrontal cortex coordinates were localized in the MFG in 87.5% of cases (seven out of eight cases) and in the PCG in one case (Table 4). The distance between individual fMRI derived and group-level coordinates (Herwig et al., 2003; Cotelli et al., 2010; Fox et al., 2013) was significant at the 12 mm threshold only for the BA9 (*p* = 0.039) and BA46 (*p* = 0.004) coordinates, the latter reaching significance also with the lenient threshold (*p* = 0.004; Table 4). No other significant differences emerged (all *p*’s > 0.47).

### Validation With Seed-Connectivity Analysis

Table 5 shows the correspondence between the individual ICA maps of the DMN and FPN and the seed connectivity maps obtained using individual rs-fMRI targets or the group-level coordinates as seeds. The template matching procedure revealed that the IPL individual targets matched the ICA-derived DMN individual map in all cases (100%), while the group-level targets matched the individual DMN maps only in 62% (Herwig et al., 2003) and 31% (Cotelli et al., 2012) of cases. For the DLPFC FPN target, the tailored fMRI-based targets showed a high correspondence with the ICA-derived FPN network (85% of cases), while correspondence was lower for the other group-level targets (matching in 69% of cases for the DLPFC BA9 target, 54% for DLPFC 5 cm-rule, 38% for the other DLPFC targets).

## Discussion

The combination of neuroimaging and neurostimulation techniques to design personalized protocols is an emerging research field, which may enhance the precision of rTMS (Cocchi and Zalesky, 2018). Here, we tested the feasibility of a tailored network-based rTMS protocol in a sample of AD patients, showing how to target AD relevant networks by extracting their hub coordinates from individual rs-fMRI.

The advantages of the proposed method over previous approaches become clear when the spatial extent of TMS-induced activation is considered. Although TMS focality is difficult to estimate because of technical and anatomical factors (Thielscher and Kammer, 2004), computational models (Fox et al., 2013) indicate a physiological response to TMS within a spatial extent of 12 mm when considering the mostly used standard figure-of-eight coil. Our comparisons revealed a significant distance between functionally defined individual targets and anatomical group-level coordinates when assuming a stimulation field size <12 mm, thus favoring the spatial selectivity of our approach. This advantage is even more striking in the hypothesis that rTMS focality is <2 mm, as suggested by a recent study recording single-unit activity in the parietal cortex of rhesus monkeys (Romero et al., 2019). Even assuming a larger (e.g., 20 mm) focality for TMS, the proposed approach has important advantages. While at a 20 mm threshold the distance between individual and traditional coordinates might not exceed TMS focality, we observed a loss of precision in targeting. Indeed, the sensitivity of group-level coordinates was 54–62% at most, indicating that in 46–38% of cases other networks will be stimulated. Moreover, the selectivity of group-level coordinates was generally low, indicating that group-level coordinates would result in stimulation of both networks rather than in the selective targeting of the intended network. The best trade-off between sensitivity/selectivity was provided by the 5 cm rule for the DLPFC node (54–71%), however, these values are far less precise than our approach, which was designed to provide a sensitivity/selectivity of 100%.

The individual rs-fMRI targets of healthy elderly controls showed similar inter-subjects variability to that observed in AD patients for the IPL node of the DMN, whereas the tailored FPN targets showed lower variability in the group of healthy elderly controls. When considering the distance between functionally defined individual targets and group-level coordinates, results were similar in AD patients and healthy elderly controls for the DMN for both the conservative (i.e., 12 mm) and lenient (i.e., 20 mm) threshold. For the FPN targets, results were similar between groups only for the BA9 and BA46 areas, due to a lower variability in DLPFC coordinates between control subjects. These results suggest that the proposed approach may be advantageous in pathological aging, and even in healthy aging when targeting the DMN. The large variability observed between subjects’ spatial maps and across individual targets is consistent with the knowledge that the brain’s structure and function undergo substantial changes both in physiological aging and in AD, with a massive networks’ reorganization (Dubovik et al., 2013; Edde et al., 2020; He et al., 2020; Pläschke et al., 2020). Our data suggest that this reorganization may be more pronounced in pathological than physiological aging, accounting for the remarkable importance of an individual targeting approach in this latter population.

Bearing this in mind, going beyond an anatomical approach might reveal crucial to increase rTMS clinical efficacy in patients. In our sample, the functional targets did not correspond to the expected anatomical region in 23–46% of cases, confirming a lack of function-anatomical correspondence that might explain the feeble evidence regarding clinical efficacy of rTMS in AD population. Consistently with this view, recent studies in depression showed that the efficacy of rTMS was higher when the target was selected on the basis of functional connectivity (Weigand et al., 2018; Cash et al., 2020).

The seed-connectivity analysis demonstrated the validity of our approach: seed-derived maps corresponded to the individual ICA maps obtained with our rs-fMRI tailored approach in 85–100% of cases. Moreover, this analysis confirmed the superiority of the proposed procedure compared to traditional group-level approaches, which showed a correspondence with individual maps in 70% of cases at best.

Notably, the proposed approach is not specific for a given TMS technique or protocol. Specifically, our strategy can be applied to both rTMS and theta burst stimulation techniques, and is not dependent on the type of stimulation protocol (i.e., inhibitory vs. excitatory). The choice of the type of stimulation to be delivered, while representing a key step in the design of TMS interventions, is outside the scope of this report. Here, we point out that TMS protocols for AD should take into account not only the localization of the target, but also the connectivity pattern (i.e., reduced vs. increased connectivity), the degree of pathology (i.e., affected vs. spared regions), and their interaction.

Furthermore, this approach was meant to be easily translated to other dementias and diseases affected by network dysfunction in order to design TMS disorder-specific protocols. Neurodegenerative and psychiatric diseases characterized by emotional and behavioral deficits such as the behavioral variant of frontotemporal dementia (Zhou et al., 2010) and borderline personality disorder (Quattrini et al., 2019) might benefit from stimulation of the DMN and salience network, while conditions characterized by language disturbances such as primary progressive aphasia may be suited for stimulation of the language network (Ficek et al., 2019), whereas motor disorders such as Parkinson’s disease may benefit from stimulation of the sensorimotor network (Göttlich et al., 2013).

Some possible limitations of the proposed approach should be mentioned. To be clinically usable, individualized coordinate extraction from rs-fMRI needs to be reliable. This requires (i) the definition of standard pre-processing procedure and (ii) that networks are reliable. For the first issue, while our procedure is relatively straightforward, it requires independent validation. Moreover, while we used ICA, seed-correlation analysis is a valid alternative that has already been applied in other studies (Nilakantan et al., 2019). Seed-based approaches typically use the hippocampus as seed region to derive the DMN parietal node, defined as the most functionally correlated region. While we used a different strategy (based on the local cluster maxima) that does not provide information on the strength of the correlation with the hippocampus or other DMN regions, our approach extracted the region most involved and active within the DMN component. Moreover, one advantage of ICA-based compared to seed-based approaches is that they enable to extract statistically independent sources, while the latter cannot distinguish whether a brain region is shared by multiple networks.

Furthermore, in our study we used relatively advanced fMRI sequences that may not be available at all centers (multiband, 600 volumes, 2 mm voxel resolution, TR = 1000 ms). Future studies might find appropriate to investigate whether this approach can be translated to other scanners and rs-fMRI protocols. For the second aspect, in our study we counterbalanced this issue by extracting the network 10-fold and ensuring that the same component was extracted reliably. Several automated tools are available to assess networks reliability (e.g., ICASSO; Himberg et al., 2004) and the use of these tools is recommended to ensure that the extracted networks are stable enough for rTMS targeting. Finally, while we might expect our approach to increase rTMS efficacy by increasing the precision of target localization, this was not formally tested and was not the objective of the present study. Forthcoming studies testing the differential impact of network-based versus traditional approaches on relevant clinical outcomes, such as memory performance, are needed to directly test this assumption.

In conclusion, based on a functional network perspective, we proposed a procedure for individual identification of TMS targets, paving the way for unprecedented personalized connectivity-based rTMS treatments for AD.

## Data Availability Statement

The datasets presented in this study can be found in online repositories. The names of the repository/repositories and accession number(s) can be found below: 10.17632/5zxyrvc5nz.2.

## Ethics Statement

The studies involving human participants were reviewed and approved by the Local Ethics Committee of IRCCS Istituto Centro San Giovanni di Dio Fatebenefratelli, Brescia, Italy. The patients/participants provided their written informed consent to participate in this study.

## Author Contributions

CB, DB, SB, and MP designed the research. CB, DB, GQ, RG, and MP performed the research. GQ and MP analyzed the data. CB, DB, and MP drafted the article. SB, GQ, and RG revised the article critically. All authors contributed to the article and approved the submitted version.

## Conflict of Interest

The authors declare that the research was conducted in the absence of any commercial or financial relationships that could be construed as a potential conflict of interest.

## Footnotes

1 http://www.fmrib.ox.ac.uk/fsl/

2 https://fsl.fmrib.ox.ac.uk/fsl/fslwiki/MELODIC
